# Detection of Human Bocavirus mRNA in Respiratory Secretions Correlates with High Viral Load and Concurrent Diarrhea

**DOI:** 10.1371/journal.pone.0021083

**Published:** 2011-06-20

**Authors:** José Luiz Proença-Modena, Talita Bianca Gagliardi, Flávia Escremim de Paula, Marisa Akiko Iwamoto, Miriã Ferreira Criado, Ataíde A. Camara, Gustavo Olszanski Acrani, Otávio Augusto Leite Cintra, Maria Célia Cervi, Luisa Karla de Paula Arruda, Eurico Arruda

**Affiliations:** 1 Department of Cell Biology, University of São Paulo School of Medicine, Ribeirão Preto, São Paulo, Brazil; 2 Department of Internal Medicine, University of São Paulo School of Medicine, Ribeirão Preto, São Paulo, Brazil; 3 Department of Pediatrics, University of São Paulo School of Medicine, Ribeirão Preto, São Paulo, Brazil; 4 Virology Research Center, University of São Paulo School of Medicine, Ribeirão Preto, São Paulo, Brazil; 5 Santa Lydia Hospital, Ribeirão Preto, São Paulo, Brazil; University of Texas Medical Branch, United States of America

## Abstract

Human bocavirus (HBoV) is a parvovirus recently identified in association with acute respiratory infections (ARI). Despite its worldwide occurrence, little is known on the pathogenesis of HBoV infections. In addition, few systematic studies of HBoV in ARI have been conducted in Latin America. Therefore, in order to test whether active viral replication of human bocavirus is associated with respiratory diseases and to understand the clinical impact of this virus in patients with these diseases, we performed a 3-year retrospective hospital-based study of HBoV in outpatients and inpatients with symptoms of Acute Respiratory Infections (ARI) in Brazil. Nasopharyngeal aspirates (NPAs) from 1015 patients with respiratory symptoms were tested for HBoV DNA by PCR. All samples positive for HBoV were tested by PCR for all other respiratory viruses, had HBoV viral loads determined by quantitative real time PCR and, when possible, were tested by RT-PCR for HBoV VP1 mRNA, as evidence of active viral replication. HBoV was detected in 4.8% of patients, with annual rates of 10.0%, 3.0% and 3.0% in 2005, 2006 and 2007, respectively. The range of respiratory symptoms was similar between HBoV-positive and HBoV-negative ARI patients. However, a higher rate of diarrhea was observed in HBoV-positive patients. High HBoV viral loads (>10^8^ copies/mL) and diarrhea were significantly more frequent in patients with exclusive infection by HBoV and in patients with detection of HBoV VP1 mRNA than in patients with viral co-infection, detected in 72.9% of patients with HBoV. In summary, our data demonstrated that active HBoV replication was detected in a small percentage of patients with ARI and was correlated with concurrent diarrhea and lack of other viral co-infections.

## Introduction

The discovery of human bocavirus (HBoV) was the result of a viral metagenomic study of respiratory secretions from Swedish children with symptoms of acute respiratory infection (ARI) reported in 2005 [1]. HBoV is classified in the family *Parvoviridae*, which includes small non-enveloped, icosahedral viruses with 5.3 kb single-stranded DNA genome containing three open reading frames (ORFs). The first two sequential ORFs encode non-structural proteins NS1 and NP-1, whereas the third downstream ORF encodes two viral capsid proteins, VP1 and VP2 [1]. HBoV circulates worldwide [2] and more recent phylogenetic analyses revealed that three novel HBoV species, different from the respiratory HBoV1, named HBoV2, 3 and 4, are frequently detected in stools from children with acute gastroenteritis [3], [4], [5], [6].

HBoV is often detected in patients with ARI [7], [8], [9], including those with wheezing, croup, cough, rhinorrhea and fever [2], [10]. The most frequent clinical diagnoses associated with respiratory HBoV are general upper respiratory tract infections (URTI), bronchiolitis, pneumonia, bronchitis and exacerbation of asthma [2]. Symptoms associated with HBoV usually last 1–2 weeks [11], [12], but the agent has also been detected in association with prolonged fever [13]. An association of HBoV with respiratory disease has been based mostly on the significantly higher frequencies of detection of the agent in ARI patients than in control subjects without respiratory symptoms [8], [10], [14]. HBoV is also detected in feces from children with diarrhea with frequencies ranging from 0.8% to 9% [6], [15], [16]. In addition, HBoV DNA has been detected in tonsil tissue in children with chronic tonsillitis undergoing surgical resection [17], [18].

Although HBoV has been propagated in primary cultures of human respiratory cells [19], isolation of the agent from clinical samples has not been achieved in common cell lines. Moreover, no experimental animal model of HBoV infection has been developed, which hampers fulfillment of Koch's postulates for a definitive association of HBoV with disease. This scenario is further complicated by the possibility that HBoV may persist in a way similar to other viruses of the family *Parvoviridae* [20], with consequent prolonged detection in human secretions. In addition, detection of HBoV DNA by PCR is commonly associated with detection of genomes of other viruses whose roles in pathogenesis of human diseases is already established [21].

This article reports a cross-sectional study of HBoV in ARI patients from Ribeirão Preto, Brazil, in which the shedding of VP1 mRNA in respiratory secretions was used as surrogate marker for active HBoV replication, to look for correlations with viral load, and presence of particular clinical manifestations and simultaneous detection of other respiratory viruses.

## Results

### Frequency and over time distribution of HBoV detection

**Figure 1:**
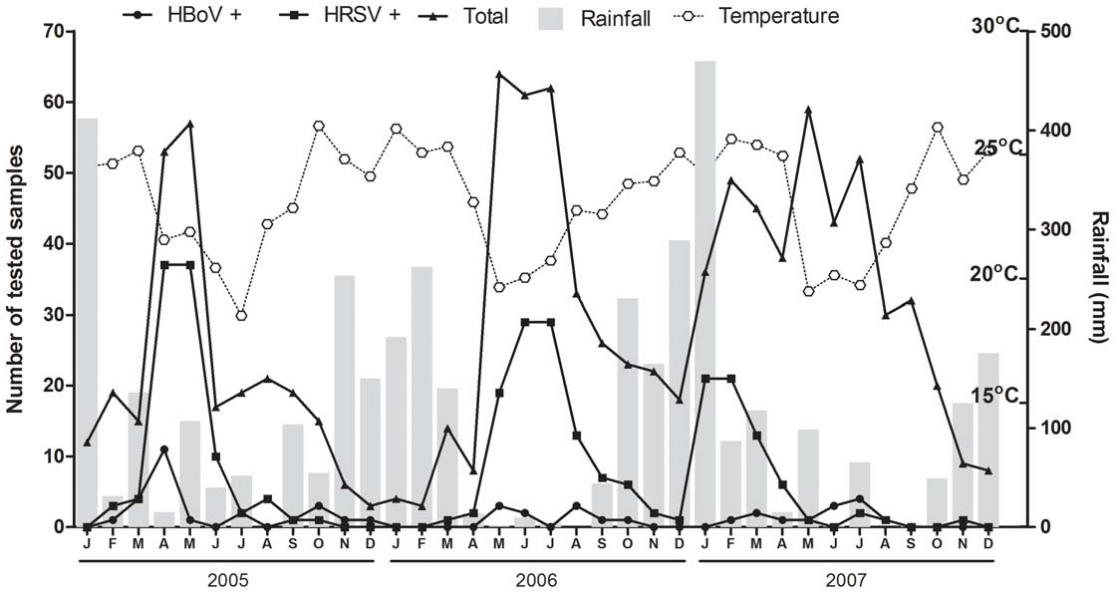
Monthly distribution of samples positive for HBoV and HRSV in the years 2005 through 2007 in Ribeirão Preto, Brazil in relation to monthly accumulated rainfall and average temperauture (http://www.ciiagro.sp.gov.br).

HBoV genome was detected by PCR in 48 out of 1015 (4.8%) respiratory samples from ARI patients. Out of the 48 HBoV positive samples, 40 (83.3%) were from patients under or equal to 2 years of age (≤2 years old) and only 2 (4.1%) were from adult patients. HBoV was detected in 2.8% of 70 adult patients enrolled in the study. The patient demographics and clinical information are summarized on Table 1. The vast majority of studied patients were under or equal to 2 years of age, in annual proportions of 93.3% (239/256) in 2005, 69.2% (234/338) in 2006, and 59.8% (252/421) in 2007. The overall frequency of HBoV detection was 10.0% in 2005 and 3.0% both in 2006 and 2007, with monthly rates that did not indicate consistent seasonality (Figure 1). It was noticeable that HBoV circulated roughly along with HRSV (Figure 1). In this 3-year study period, there was a trend for an increase in the total number of respiratory samples from children submitted for viral testing, HRSV and HBoV positivity, in association with dry season and declining average montly temperatures [22].

It is noteworthy that HBoV was not detected in stored samples obtained from 50 patients without respiratory symptoms, seen at the emergency room for reasons unrelated to the respiratory tract from 1999 to 2000 [23].

**Table 1:** Clinical and demographic data of patients with and without HBoV.

Clinical Data	Patients		
	HBoV +	HBoV -	Total
Patients	48 (4.8%)	967 (95.2%)	1015 (100.0%)
Masculine gender	32 (66.6%)	504 (52.2%)	536 (52.8%)
Age (median of months)	8.5	8	8
Cough	44 (91.6%)	843 (87.2%)	887 (87.4%)
Coryza	26 (54.1%)	507 (52.4%)	533 (52.5%)
Sneezing	8 (16.6%)	219 (22.6%)	227 (22.3%)
Fever	27 (56.2%)	636 (65.7%)	663 (65.3%)
Wheezing	20 (41.6%)	400 (41.3%)	420 (41.4%)
Dyspnea	27 (56.2%)	464 (47.9%)	491 (48.3%)
Nasal obstruction	15 (31.2%)	360 (37.2%)	375 (36.9%)
Diarrhea*	10 (20.8%)	53 (5.5%)	63 (6.3%)
Requirement for hospitalization*	40 (83.3%)	653 (67.5%)	693 (68.3%)
Length of hospital stay (median of days)	9	9	9
Requirement for O_2_	23 (47.9%)	403 (41.6%)	426 (41.9%)
Requirement for PAP	2 (4.0%)	70 (7.2%)	72 (7.1%)
ICS = 0	7 (14.6%)	219 (22.6%)	226 (22.2%)
ICS = 1	6 (12.5%)	144 (14.9%)	150 (14.8%)
ICS = 2	9 (18.8%)	179 (18.5%)	188 (18.5%)
ICS = 3	6 (12.5%)	132 (13.6%)	138 (13.6%)
ICS = 4	13 (27%)	133 (13.8%)	146 (14.4%)
ICS = 5	5 (10.4%)	104 (10.8%)	109 (10.8%)
ICS = 6	1 (2.1%)	32 (3.3%)	33 (3.3%)
ICS = 7	1 (2.1%)	24 (2.5%)	25 (2.4%)
LRTI*	41 (85.4%)	698 (72.2%)	739 (72.8%)
URTI*	7 (14.6%)	269 (27.8%)	276 (27.2%)
AOM	4 (8.3%)	64 (6.6%)	68 (6.7%)
GERD*	4 (8.3%)	41 (4.2%)	45 (4.4%)

PAP = positive airway pressure; ICS = index of clinical severity; LRTI =  Lower respiratory tract infection; URTI =  Upper respiratory tract infection; AOM =  Acute otitis media; GERD =  Gastro-esophageal reflux disease.* p<0.05.

### Clinical analyses

For the purpose of the analysis, the patients were divided into two groups: those with age equal or less than 2 years of age (410 males and 335 females; median and mean age ± SD: 5 and 6.45±5.49 months), and those older than 2 years (125 males and 145 females; 63.5 and 145.15±187.75 months). In general, the clinical findings were more severe in patients younger than 2 years (Table S2). The most frequent respiratory symptoms/signs (present in >50%) seen in HBoV-positive patients were cough, fever, dyspnea and coryza, but wheezing, nasal obstruction and sneezing were also recorded. No significant differences were noted in the frequencies of specific respiratory symptoms/signs between HBoV positive and negative patients (Table 1).

Of 1015 studied patients, 721 (71.0%) were diagnosed with LRTI and 294 (28.9%) with URTI. The complications of AOM and GERD were diagnosed in 77 (7.6%) and 44 (4.4%) patients, respectively (Table S2). Over one third of the patients had illness of low or moderate severity and 37.0% of them had an ICS = 0 or 1,and only 5.7% had ICS = 6 or 7 (Table 1).

Significantly higher frequencies of LRTI (85.4% vs. 72.2%, p = 0.01), requirement for hospitalization (83.3% vs. 67.5%, p = 0.02), and diarrhea (20.8% vs. 5.5%, p<0.01) were noted in the 48 HBoV-positive patients, in comparison with the 967 HBoV-negative ones (p<0.05) (Table 1). No significant association was noted between HBoV positivity and the presence of specific signs or symptoms (Table 1). Also, no significant differences were noted in length of hospital stay, rates of requirements for O_2_ and positive airway pressure, AOM, and illness severity as measured by ICS, between HBoV positive and negative patients (Table 1). While the frequency of GERD was roughly twice as high in HBoV-positive, than in HBoV-negative patients (p = 0.04), only 4 HBoV patients had GERD (Table 1), a low number of cases that restricts the power of the comparison.

Logistic regression analyses of the relationship between HBoV detection and disease, after adjustment for patient age, gender and concurrent HRSV infection, showed that the clinical features more significantly associated with HBoV were diarrhea (odds ratio [OR]  = 37.08; 95% confidence interval [CI]; 3.42–401.98; p<0.01) and GERD (OR  = 14.35; 95% CI, 1.03–200.5; p = 0.05). However, neither the rate of hospitalization nor the presence of LRTI were associated with HBoV detection by logistic regression (OR  = 2.14; 95% CI, 0.89–5.19; p = 0.09 and OR  = 2.15; 95% CI, 0.80–5.73; p = 0.13, respectively).

### Viral co-infections

All samples positive for HBoV by conventional PCR were tested for the other respiratory viruses, including HRSV, HRV, HMPV, FLU, HPIV, HCoV and HAdV. Of 48 HBoV-positive patients, 35 (72.9%) had at least one additional respiratory virus detected (Table S3). The viruses most frequently detected simultaneously with HBoV were HRSV in 22 (45.8%), HRV in 16 (33.3%), HMPV in 9 (18.8%), and HAdV in 7 (14.5%) patients. The frequencies of co-detection of one, two, three and four additional viruses in patients with HBoV were respectively 35.4%, 27.1%, 4.1% and 6.3% (Table S3).

To assess whether this high frequency of co-detection of other respiratory viruses was a finding specific of HBoV-infected patients, a subset of 48 samples from HBoV-negative ARI patients, collected in the same season as those from HBoV-positive ones, was randomly selected for testing by PCR for the other respiratory viruses, without knowing the result of the HRSV or influenza screening for which the samples had been originally submitted. Such strategy was adopted since it would not be possible to test all samples for all respiratory viruses. A single respiratory virus different from HBoV was detected in 26 of 48 (54.1%) patients, and co-detections with more than one agent were found in an additional 13 (27.1%) (Table S3).

There were no significant differences in frequencies of specific respiratory symptoms between the patients with ARI in whose samples only HBoV was detected, and those with HBoV and co-detection of other viruses. However, significantly lower frequencies of diarrhea (OR  = 0.08; 95% CI  = 0.01–0.40; p<0.01) and complication of GERD (OR  = 0.10; 95% CI  = 0.01–1.05; p = 0.05) were noted in patients with co-detection of other viruses in addition to HBoV (Table S4).

### Quantification of HBoV DNA and detection of HBoV mRNA

**Figure 2:**
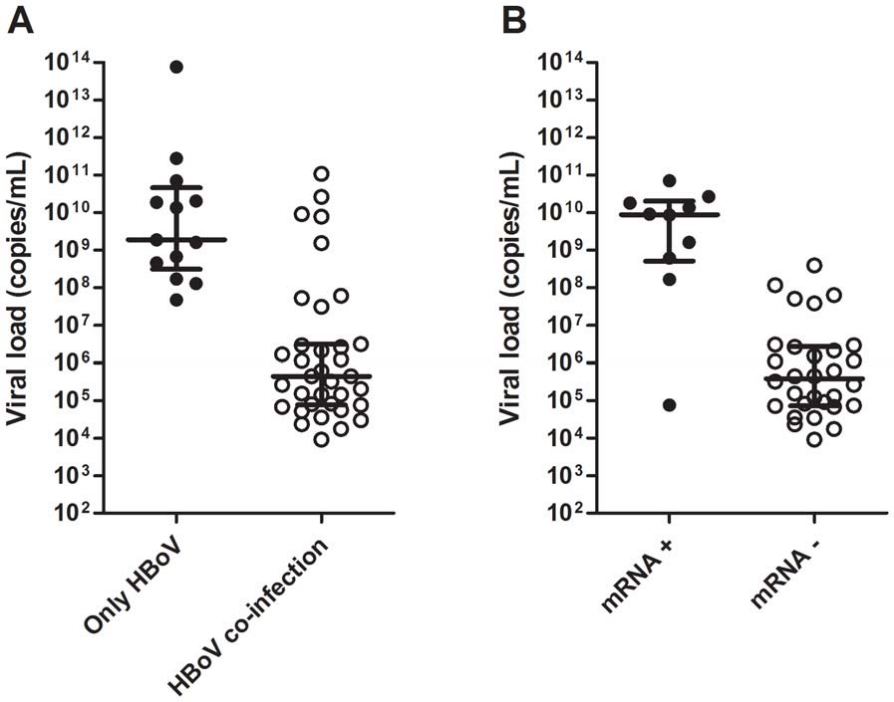
Viral loads of HBoV (copies of genomic DNA/mL) in NPAs by qPCR. **A.** HBoV viral loads in NPAs from patients with HBoV as single agent (black circles), and with simultaneous detection of other respiratory viruses (white circles). **B.** HBoV viral loads in NPAs from patients with (black circles) and without (white circles) detectable shedding of HBoV VP1 mRNA by real time PCR.

Absolute quantification of DNA by qPCR revealed that the HBoV viral load in HBoV-positive patients varied very broadly, over a range that reached 10 decimal orders of magnitude, with median value of 2.37×10^6^ copies/mL (Figure 2 and Table S5). Importantly, the median viral load in samples from patients in whom HBoV was the only virus detected (1.91 × 10^9^ copies/mL), was significantly higher than that in the patients with HBoV in co-infection with other respiratory viruses (4.41 × 10^5^ copies/mL) (p<0.001) (Figure 2a).

A comparison of demographics and clinical data between patients shedding very high HBoV viral loads (>10^8^ copies/mL of NPA) and those shedding lower loads, evidenced significantly higher frequencies of diarrhea (p<0.01) in patients shedding higher viral loads (Table 2). Remarkably, detection of other respiratory viruses simultaneously with HBoV was significantly less frequent in samples from patients shedding very high loads of HBoV (p<0.01) (Table 2). Logistic regression analyses confirmed that diarrhea (OR  = 6.53; 95% CI  = 1.41–30.26; p = 0.02) were directly related, whereas simultaneous detection of other viruses in the NPAs was inversely related (OR  = 0.01; 95% CI  = 0.00–0.13; p<0.01) to the shedding of very high HBoV loads.

**Table 2:** Clinical and demographic data of ARI patients shedding very high or lower HBoV loads.

Clinical Data	Patients		
	Shedding very high viral loads#	Shedding lower viral loads	Total
Patients	17 (35.4%)	31 (64.6%)	48 (100.0%)
Masculine gender	9 (52.9%)	23 (74.2%)	32 (66.6%)
Age (median of months)	10	8	8.5
HBoV viral load (median copies/mL)*	1.0E+09	2.66E+05	2.37E+06
Co-detection of other viruses*	5 (29.4%)	30 (96.8%)	35 (72.9%)
Cough	16 (94.1%)	28 (90.3%)	44 (91.6%)
Coryza	10 (58.8%)	16 (51.6%)	26 (54.1%)
Sneezing	4 (23.5%)	4 (12.9%)	8 (16.6%)
Fever	11 (64.8%)	16 (51.6%)	27 (56.2%)
Wheezing	7 (41.1%)	13 (41.9%)	20 (41.6%)
Dyspnea	8 (47.0%)	19 (61.3%)	27 (56.2%)
Nasal obstruction	4 (23.5%)	11 (35.5%)	15 (31.2%)
Diarrhea*	7 (41.1%)	3 (9.7%)	10 (20.8%)
Requirement for hospitalization	14 (82.3%)	26 (83.8%)	40 (83.3%)
Length of hospital stay (median of days)	8	9	9
Requirement for O_2_	7 (41.1%)	16 (51.6%)	23 (47.9%)
Requirement for PAP	0 (0.0%)	2 (6.5%)	2 (4.2%)
ICS = 0	4 (23.5%)	3 (9.7%)	7 (14.6%)
ICS = 1	1 (5.9%)	5 (16.0%)	6 (12.5%)
ICS = 2	5 (29.4%)	4 (13.0%)	9 (18.8%)
ICS = 3	0 (0.0%)	6 (19.4%)	6 (12.5%)
ICS = 4	5 (29.4%)	8 (26.0%)	13 (27.0%)
ICS = 5	2 (11.8%)	3 (9.7%)	5 (10.4%)
ICS = 6	0 (0.0%)	1 (3.2%)	1 (2.1%)
ICS = 7	0 (0.0%)	1 (3.2%)	1 (2.1%)
LRTI	13 (76.4%)	28 (90.3%)	41 (85.4%)
URTI	4 (23.5%)	3 (9.7%)	7 (14.6%)
AOM	1 (5.8%)	3 (9.7%)	4 (8.3%)
GERD	3 (17.6%)	1 (3.2%)	4 (8.3%)

# HBoV viral load >10^8^ copies/mL of NPA; ^¶^HBoV viral load <10^8^ copies/mL of NPA; PAP = positive airway pressure; ICS = index of clinical severity; LRTI =  Lower respiratory tract infection; URTI =  Upper respiratory tract infection; AOM =  Acute otitis media; GERD =  Gastro-esophageal reflux disease.* p<0.05

The association of shedding very high loads of HBoV (>10^8^ copies/mL) with lack of co-detection of other respiratory viruses suggested that such high viral loads may result from early acute HBoV infections with active viral replication, as opposed to long term shedding unrelated to current clinical symptoms. In order to confirm this hypothesis, an assay was developed to detect HBoV VP1 mRNA in NPAs by real time RT-PCR done on total RNA extracts previously treated with DNAse I. As an internal control, real time RT-PCR was done for the housekeeping gene of β-actin. When simultaneously positive for both HBoV VP1 and β-actin mRNAs, this assay was considered a marker of active HBoV replication. Positivity for HBoV VP1 or β-actin by PCR done directly on DNAse-treated material without previous reverse transcription were considered indicative of remaining genomic DNA after incomplete DNAse treatment, and samples were discarded. Of the 48 samples positive for HBoV, 40 had sufficient remaining volume left for testing. Of those, only 10 (25.0%) tested positive for VP1 mRNA and, remarkably, 9 of these had very high HBoV loads of greater than 10^8^ copies of DNA/mL. The median HBoV viral load in patients shedding VP1 mRNA was 8.85 × 10^9^ copies/mL, which was significantly higher than that in patients negative for VP1 HBoV mRNA, 3.82 ×10^5^ copies/mL (p<0.01) (Figure 2b, Table 3). The association of high viral loads with shedding of HBoV VP1 mRNA was confirmed by logistic regression (OR  = 126.0; 95% CI  = 10.19–999.99; p<0.01).

**Table 3:** Clinical and demographic data of patients shedding HBoV VP1 mRNA.

Clinical Data	Patients		
	RNAm +	RNAm -	Total
Patients	10 (25.0%)	30 (75.0%)	40 (100%)
Masculine gender	5 (50.0%)	21 (70%)	26 (65%)
Age (median of months)	9.5	5	8
Patients with high HBoV load#*	9 (90.0%)	2 (6.6%)	11 (27.5%)
HBoV viral load (median of copies/mL of NPA)*	8.85E+09	3.82E+05	1.13E+06
Detection of other respiratory viruses*	4 (40.0%)	28 (93.3%)	32 (80.0%)
Cough	9 (90.0%)	27 (90.0%)	36 (90.0%)
Coryza	6 (60.0%)	17 (56.6%)	23 (57.5%)
Sneezing	1 (10.0%)	5 (16.6%)	6 (15.0%)
Fever	5 (50.0%)	16 (53.3%)	21 (52.5%)
Wheezing	4 (40.0%)	11 (36.6%)	15 (37.5%)
Dyspnea	4 (40.0%)	18 (60.0%)	22 (55.0%)
Nasal obstruction	4 (40.0%)	10 (33.3%)	14 (35.0%)
Diarrhea*	4 (40.0%)	3 (10.0%)	7 (17.5%)
Requirement for hospitalization	7 (70.0%)	23 (76.6%)	30 (75.0%)
Length of hospital stay (median of days)	6	9	9
Requirement for O_2_	3 (30.0%)	16 (53.3%)	19 (47.5%)
Requirement for PAP	0 (0.0%)	2 (6.6%)	2 (5.0%)
ICS = 0	4 (40.0%)	3 (10.0%)	7 (17.5%)
ICS = 1	1 (10.0%)	5 (16.6%)	6 (15.0%)
ICS = 2	2 (20.0%)	4 (13.4%)	6 (15.0%)
ICS = 3	0 (0.0%)	5 (16.6%)	5 (12.5%)
ICS = 4	2 (20.0%)	8 (26.8%)	10 (25.0%)
ICS = 5	1 (10.0%)	3 (10.0%)	4 (10.0%)
ICS = 6	0 (0.0%)	1 (3.3%)	1 (2.5%)
ICS = 7	0 (0.0%)	1 (3.3%)	1 (2.5%)
LRTI*	6 (60.0%)	28 (93.3%)	34 (77.5%)
URTI*	4 (40.0%)	2 (6.6%)	6 (15.0%)
AOM	2 (20.0%)	2 (6.6%)	4 (10.0%)
GERD	1 (10.0%)	3 (10.0%)	4 (10.0%)

# HBoV viral load >10^8^ copies/mL of NPA, PAP = positive airway pressure; ICS = index of clinical severity; LRTI =  Lower respiratory tract infection; URTI =  Upper respiratory tract infection; AOM =  Acute otitis media; GERD =  Gastro-esophageal reflux disease.* p<0.05.

Importantly, a significant inverse association was noted between shedding of HBoV VP1 mRNA and having another respiratory virus detected in the NPA. Four of 10 (40.0%) patients who shed HBoV VP1 mRNA also had another respiratory virus detected in their NPAs, whereas 28 of 30 (93%) of the patients in whose samples shedding of VP1 mRNA was not present, had another respiratory virus detected in their NPAs (p<0.01) (Table 3). Such inverse association was confirmed by logistic regression analysis (OR  = 0.05; 95% CI  = 0.01–0.32; p<0.01).

A comparison of clinical features between HBoV patients with and without shedding of VP1 mRNA revealed an association of active viral replication with diarrhea (OR  = 6.00; 95% CI  = 1.15–34.14; p = 0.04) and a clinical diagnosis of URTI (OR  = 19.33; 95% CI  = 1.82–294.96; p = 0.01). The frequencies of diarrhea and diagnosis of URTI in patients shedding VP1 mRNA were 40%, while in patients without detectable VP1 mRNA they were respectively 10% and 6.6% (Table 3).

## Discussion

The results of this cross-sectional study of HBoV in ARI patients from Ribeirão Preto, Brazil, indicate that shedding of VP1 mRNA in respiratory secretions, as a marker of HBoV replication, correlates positively with high viral load, presence of diarrhea, and lack of co-infection by other respiratory viruses.

During the last five or six years since its discovery, HBoV has been detected in association with respiratory and gastrointestinal infections, mostly of children, in many countries in all continents [2], [24]. Rates of detection of HBoV in ARI studies have varied from 1.5% to 19.0%, depending on patient age, case definition, and geographic location [2], [24]. In the present study, the overall detection rate of HBoV in ARI patients was 4.8%, similar to rates in other regions of the world. However, it should be kept in mind that this study, like most others published on HBoV, was a hospital-based surveillance, subject to selection bias. Therefore, rates of HBoV in ARI in the community at large could be different.

To the best of our knowledge, this has been the largest study of HBoV in ARI conducted in Brazil and South America. A few previous studies conducted in the region reported HBoV infection rates of 6.0% to 10.7% in patients with ARI [9], [25], [26], [27] and in 2.0% of patients with gastroenteritis [15]. Importantly, the present study revealed a significantly higher rate of concurrent diarrhea in patients with ARI positive for HBoV as compared to those with ARI but without detectable HBoV, suggesting a causative role for HBoV in gastrointestinal manifestations observed in ARI patients. In general, parvoviruses replicate more efficiently in tissues with high cellular turnover, such as respiratory and digestive epithelia [28]. Several members of the family *Parvoviridae* are known to cause gastroenteritis, such as canine parvovirus (CPV), feline panleukopenia virus (FPV) and canine minute virus (CnMV). In addition studies previously published by others have reported HBoV detection in stools from patients with gastroenteritis [29]. Although the mechanisms of pathogenesis and routes of infection of HBoV are not yet understood, some information is available for bovine parvovirus (BPV), another member of the genus *Bocavirus*. BPV causes respiratory and enteric diseases in calves following initial replication in the tonsils and intestinal epithelium, from where it spreads into the blood, lymphoid tissues and respiratory epithelium. BPV induces atrophy of microvilli in the small intestine and death (by apoptosis and necrosis) of lymphoid cells and ciliated cells of the respiratory epithelium [28].

It is interesting that novel species of HBoV (HBoV 2, 3 and 4) associated with gastrointestinal infections have recently been identified [3], [4], [5] and two of those have been detected in stools from Brazilian patients with acute diarrhea, at rates varying from 0.6% for HBoV 3 to 20.8% for HBoV 2 [6]. However, a causative role for HBoV in human diarrhea still awaits confirmation [30].

Interestingly, in this study a trend was noted for an increase in respiratory infections, positivity for HRSV and HBoV, in association with drier and cooler months. While the reasons for this are not entirely understood, one possibility is that the increased respiratory virus activity in March and April could be favored by the initiation of school term one or two months before, when assembly of susceptible children could provide conditions for viral spread. The frequency of detection of HBoV in 2005 was greater than three times that observed in the two following years, evidence that circulation of this agent in this region of Brazil may vary greatly from year to year, in a way similar to that previously documented in Italy [31].

Even though HBoV has been regarded as an infectious agent present worldwide, its pathogenic role in respiratory illness is still debatable. This virus is frequently detected in co-infection with other respiratory viruses of well-established pathogenic role [2], [24]. In addition, HBoV is fastidious to propagate in conventional cell cultures and an experimental infection model is lacking, what limits advances in understanding pathogenesis. Therefore, the search for an association of HBoV infections with possible molecular markers of active viral replication is a useful approache to investigate pathogenesis of this emerging virus.

In the present study, HBoV DNA was detected by PCR in samples from ARI patients, but not in nasopharyngeal secretions collected from asymptomatic children approximately 5 years before (1999–2000) in the same hospital. The significance of this finding is limited by the relatively small number of control samples available for testing, and by the fact that they were collected years before. However, the lack of detection of HBoV DNA by a sensitive PCR assay in samples collected from children carefully scrutinized for the presence of respiratory symptoms, adds support for a pathogenic role of HBoV in ARI, in agreement with previous studies [20], [32], [33].

High frequencies of co-infection by other respiratory viruses in patients with ARI and HBoV were confirmed in this study with a rate of 72.9% (35 of 48) of co-infections, mostly caused by HRSV, HRV, HMPV and HAdV, and frequently by more than one additional agent. This was similar to the 75% reported by Allander et al., in Sweden [10], and higher than the 50% reported by Esposito et al., in Italy [34]. While viral co-infections have long been recognized in ARI studies, the advent of highly sensitive PCR assays magnified their observation. In the particular case of HBoV, frequencies of co-infections have consistently been reported to be higher than those observed for other respiratory infections, reaching rates similar to those reported for HAdV [35], a tendency confirmed by the present study. Yet unproven, this may suggest that long term shedding of HBoV DNA, unrelated to current illness may regularly occur, as it is known to happen with HAdV [36]. Indeed, HBoV DNA was found by PCR in lymphocytes from palatine tonsils of 32% of children undergoing tonsillectomy [18], suggesting that this virus can establish latent or persistent infection in pharyngeal lymphoid tissue.

Another important piece of information befitting a systemic nature of HBoV infections is the finding of viremia, reported by Tozer et al. in children with ARI [37]. Viremia was also reported by Allander et al [10] in HBoV-exclusive infections, a feature strongly suggestive of a causal role for this agent in acute symptomatic infections. In addition, those authors reported that patients with HBoV without concurrent infections by other respiratory viruses, tended to shed higher HBoV loads [10]. In this regard, an association of high viral loads in NPAs with HBoV acute infections, as evidenced by IgM seroconversion in wheezing children, was clearly documented in a study conducted in Finland by Söderlund-Venermo [38].

These results prompted us to determine HBoV loads by qPCR in all HBoV-positive NPAs of the study. The results revealed HBoV viral loads to be significantly higher in patients without other viral co-infections than in those with multiple viruses detected. Moreover, rates of diarrhea were significantly higher in patients with very high HBoV viral loads in their NPAs. Such finding, in the absence of respiratory viral co-infections, suggests a causal role for HBoV in the genesis of the diseases presented by these patients. Nevertheless, detection of a direct evidence of active replication of HBoV in the secretions from patients was required to strengthen the causality link between the virus and disease. In order to do that, an assay to detect HBoV VP1 mRNA by real time RT-PCR in nasopharyngeal samples was developed. Production of VP1 mRNA is unequivocal evidence of HBoV replication, but its shedding in nasopharyngeal secretions does not indicate the possible sources of virus production. Because RNA is very highly unstable in ribonuclease-rich respiratory secretions, one can speculate that VP1 mRNA more likely originates from disruption of sloughed whole HBoV-infected epithelial cells directly into the RNase-inhibiting extraction reagent. Unfortunately, preparation of cytology slides that would have enabled detection of HBoV on sloughed cells by in situ hybridization was not possible given the nature of the study.

The presence of extremely high viral loads of HBoV in secretions, argues in favor of abundant sources of virus, either on or adjoining the mucosal surfaces. This observation alone highlights the extremely productive replication levels reached by HBoV in the permissive upper respiratory tract. However, in situ studies will be needed to provide definitive localization of viral replication sites.

To the best of our knowledge this is the first report of an assay for mRNA to confirm replication of HBoV in clinical samples, and its application revealed that shedding of VP1 mRNA in nasopharyngeal secretions was associated with high viral loads, presence of concurrent diarrhea, and lack of demonstrable co-infections by other respiratory viruses. Conversely, in the absence of a marker of current viral replication, HBoV was also often detected in nasopharyngeal secretions, but in much lower viral loads and frequently associated with shedding of other pathogenic respiratory viruses. It should be pointed out that VP1 mRNA was found only in 25% of the HBoV infected ARI patients, with a significant part of whom with diarrhea. This may be interpreted to mean that only one quarter of the HBoV respiratory infections are associated with genuinely acute viral replication, likely involving the gastrointestinal tract. Conversely, in most cases of HBoV detection in respiratory secretions, it may be the result of viral persistence/latency, with co-detection of another viral agent.

## Methods

### Patients and sample collection

A retrospective cross-sectional study was carried out with nasopharyngeal aspirates (NPAs) collected from 1015 patients with ARI (535 males and 480 females) with 1 month to 66 years of age (median age of 8 months). The samples were from outpatients and inpatients brought for health care at the Hospital of the University of São Paulo School of Medicine, Ribeirão Preto, SP, Brazil, from January 2005 through December 2007. The study included all samples from patients with symptoms of ARI seen at any of the hospital clinics and wards, submitted to the virology laboratory for testing. The vast majority of the samples had been originally sent to the laboratory for respiratory syncytial virus and influenza screening and no exclusion criteria were applied. Clinical information obtained retrospectively from patient records upon discharge was entered on study forms. Clinical data collected included the presence of wheezing, cough, fever, sneezing, dyspnea, coryza, nasal obstruction, diarrhea, requirement for oxygen therapy and length of hospitalization. NPAs were collected in saline solution from all patients [9] and sent on ice to the virology laboratory within four hours. In addition to the 1015 samples from ARI patients, a convenience set of 50 control samples from patients without any respiratory symptoms who were participants in a previous study [23] were used as negative controls. This group consisted of 26 males, with median age of 19.5 months, mean of 37 months (SD  = 44 months). After routine virology sample processing, remaining samples were split into backup aliquots, including two in Trizol Reagent (Invitrogen Carlsbad, CA), which were kept in storage at −70°C [9]. This study was approved by the Internal Review Board of the Hospital das Clínicas de Ribeirão Preto (HCFMRP-USP) under approval number 4856/2004. All patients or the parents or guardians of the patients in the study signed an informed consent form.

### Clinical Illness Stratification

Illnesses presented by all patients in this study were classified as lower respiratory tract infections (LRTI) and upper respiratory tract infections (URTI), with or without acute otitis media (AOM) and gastro-esophageal reflux disease (GERD). Patients with URTI had only symptoms of the upper respiratory tract, such as coryza, sneezing, nasal obstruction and sore throat, without wheezing, and dyspnea, with or without fever. Patients with LRTI had diagnosis of bronchiolitis, bronchiolitis obliterans, bronchodysplasia or pneumonia. They had wheezing and/or dyspnea, with or without fever, cough, coryza, sneezing or nasal obstruction. All patients were assigned to a single clinical definition, and could not be allocated to more than one group.

In order to assess disease severity, a numeric index of clinical severity (ICS) was adapted from Walsh et al (1997) [39], based on the presence of clear-cut criteria that indicate higher risk for severe disease: wheezing, requirement for hospitalization, oxygen therapy and positive airway pressure. For all patients the ICS was obtained by attributing 1 point each for the presence of wheezing, requirement for hospitalization, length of hospital stay longer than 5 days, requirement for O_2_, and use of O_2_ for longer than 5 days; and 2 extra points attributed in case of requirement for positive airway pressure, for a maximum ICS equal to 7.

### PCR for respiratory viruses

DNA and RNA were extracted from 250 µL of NPA using, respectively, Wizard® Genomic DNA Purification Kit (Promega®, Madison, WI, USA) and Trizol® (Invitrogen®, Carlsbad, CA, USA), following manufacturers protocol.

Detection of HBoV and other respiratory viruses was conducted by conventional PCR, as previously described [9]. For detection of HBoV and human adenovirus (HAdV), 100 ng of extracted DNA was used, whereas for other respiratory viruses, including human respiratory syncytial virus (HRSV), human metapneumovirus (HMPV), human rhinovirus (HRV), human influenza virus (FLU), human parainfluenza viruses (HPIV) and human coronaviruses (HCoV), PCR was done with cDNA generated by reverse transcription (RT) of 1 µg of RNA, primed with 10 pmol of random hexamers, using ImProm-II Reverse Transcriptase (Promega, Madison, WI) following manufacturer's instructions. Prior to reverse transcription, RNA was denatured at 95°C for 5 minutes, then immediately placed on ice while reverse transcriptase was added and then incubated at 42°C for 2 hours. PCR was done with150 ng of DNA, 10 pmol forward and reverse primers (Table S1), 50 mM MgCL_2_, 10 µM deoxynucleoside triphosphate (dNTP), 2.5 µL of supplied 10 X buffer, 1U of *Taq* DNA polymerase (Invitrogen Carlsbad, CA) and water to complete the volume of 25 µL. The cycling conditions were denaturation at 95°C for 5 min, followed by 35 cycles of 94°C for 1 min, 48°C to 54°C for 1 min, 72°C for 2 min, and then a final extension step of 72°C for 10 min [9]. All PCR products were analyzed by electrophoresis in ethidium bromide-stained 1.5% agarose gels. PCR products of all tested viruses were cloned into the plasmid pGEM-T easy (Promega, Madison, Wisconsin) and used to determine detection limits for each assay. All clones were sequenced with BigDye Terminator v3.1 Cycle Sequencing (Applied Biosystems, Foster City, CA) using the ABI Prism 3100 Genetic Analyzer. The detection limits for the PCR assays were determined by testing serial decimal dilutions of the clones, and results varied from 1 to 100 copies of plasmid DNA. The limit of detection for HBoV was determined to be 40 copies. All applicable measures to prevent contamination of PCR reactions were taken in this study.

### Quantitative real-time PCR for HBoV

The quantitative PCR (qPCR) for HBoV was targeted to the viral NP1 gene according to previously published procedures [40]. Briefly, the reaction was performed in triplicate in a final volume of 10 µL, containing 150 ng of template DNA, 5 µL of TaqMan universal PCR master mix (Applied Biosystems Foster City, CA), 300 nmol/L of each primer and 150 nmol/L of probe (Table S1). The amplification was performed on a 7300 Real Time PCR system (Applied Biosystems Foster City, CA) with the following settings: 50°C for 10 min, 95°C for 3 min and 45 cycles of 95°C for 15 s and 60°C for 1 min. A standard curve of amplification was produced using serial decimal dilutions of a plasmid (pGEM-FULL4HBoV) in which a fragment of the HBoV NP1 gene (2270 nt to 3280 nt) was cloned. A positive qPCR for HBoV was considered when the threshold was reached before the 40^th^ cycle with fluorescence count higher than 0.5. The detection limit was 4.6 copies of HBoV plasmid, the slope and the R2 of the standard curve were −3,386 and 0,994, respectively. All qPCR results were normalized by the amplification of the β-actin gene, included in duplicate in all tested batches, using 300 nmol/L of each primer and 150 nmol/L of the probe (Table S1). With this approach, viral loads were determined as the number of copies of HBoV DNA per mL of NPAs.

### Real-time RT-PCR for the VP1 mRNA of HBoV

Shedding of HBoV VP1 mRNA was tested by real-time RT-PCR in NPAs to ascertain the presence of active viral replication, as opposed to shedding of viral genome. To ensure target-specific amplification total RNA extraction products were treated with DNAse I (Invitrogen Carlsbad, CA) 2 h prior to PCR. As a control, the same PCR was done in parallel directly with the RNA extraction product without previous RT. Samples were considered positive for HBoV VP1 mRNA only when they were simultaneously negative for amplification directly from the extracted RNA without previous RT. RT was performed on 1 µg of RNA extracted by Trizol using10 pmol of random primers, according to the protocol provided by the manufacturer. After RNA denaturation at 95°C for 5 min reverse transcriptase was added and incubation went on for 2 h at 42°C. PCR was then carried out with 150 ng of cDNA, with 300 nmol/L of each primer (VP1-F and VP2-R), 150 nmol/L of the probe (VP1-S, Table S1), and 5 µL of TaqMan universal PCR master mix (Applied Biosystems Foster City, CA). Amplification was done using the 7300 Real Time PCR system (Applied Biosystems Foster City, CA) with the following settings: 50°C for 10 min, 95°C for 3 min and 45cycles of 95°C for 15 s and 60°C for 1 m. All samples, including all cDNAs and RNAs pre-treated with DNAse, were tested by qPCR for β-actin mRNA, following the protocol described above. Samples found to be positive for β-actin by amplification directly from the RNA not pre-treated with DNase, were considered to be false positives. As above mentioned, positive reactions were considered when the threshold was reached before the 40^th^ cycle, with a fluorescence higher than 0.5. The detection limit of this assay was found to be 3 copies of DNA, as determined using the decimal serial dilutions of viral cDNA obtained from in vitro transcription of a plasmid containing theVP1 gene, according to manufactures' instructions (Riboprobe in vitro transcription systems, Promega Madison, WI).

### Statistical Analysis

Correlation between positivity for HBoV and clinical conditions was assessed by the Chi-square test. Statistical analysis was conducted using the BioEstat 3.0 software (CNPq, Brazil). Logistic regression was done to examine the relationship of HBoV shedding (including positivity for HBoV DNA, HBoV loads, lack of other viral co-infections and detection of HBoV VP1 mRNA) and clinical data adjusted for age, gender and detection of HRSV in the same samples [41]. To analyze the HBoV loads obtained by qPCR, data in two unpaired groups of patients were compared with the Mann-Whitney test. These analyses were performed using the *SAS*® *9.0*software (SAS Institute Inc, Raleigh, NC). A p value of ≤0.05 was chosen for significance.

## Supporting Information

Table S1 Primers and probes used in conventional and real-time PCR for respiratory viruses and β-actin.
(DOC)

Table S2 Clinical and demographic data of ARI patients
(DOC)

Table S3 Frequencies of detection of other respiratory viruses in HBoV positive and negative samples.
(DOC)

Table S4 Clinical and demographic data of patients in whom HBoV was detected alone or simultaneously with other respiratory viruses.
(DOC)

Table S5 HBoV loads determined by real time PCR in nasopharyngeal aspirates.
(DOC)
