# Supplementary material for: Detection of Human Bocavirus mRNA in Respiratory Secretions Correlates with High Viral Load and Concurrent Diarrhea
Source: PLoS One. 2011 Jun 20;6(6):e21083. doi: 10.1371/journal.pone.0021083 (PMC3118811; doi:10.1371/journal.pone.0021083)
Supplement: Table S1 — Primers and probes used in conventional and real-time PCR for respiratory viruses and β-actin. (DOC) [file pone.0021083.s001.doc]

**Table S1.** Primers and probes used in conventional and real-time PCR for respiratory viruses and β-actin.

| **Virus** | **Primer** | **Sequence (5’-3’)** |  | **Ref** |
| --- | --- | --- | --- | --- |
|  |  |  |  |  |
| HBoV | 188F | GAGCTCTGTAAGTACTATTAC |  | [1] |
|  | 542R | CTCTGTGTTGACTGAATACAG |  | [1] |
|  |  |  |  |  |
| HRSV | FV | GTTATGACACTGGTATACCAACC |  | [2] |
|  | GAB | YCAYTTTGAAGTGTTCAACTT |  | [3] |
|  | F1AB | CAACTCCATTGTTATTTGCC |  | [4] |
|  |  |  |  |  |
| HMPV | FF1 | GAGCAAATTGAAAATCCCAGACA |  | [5] |
|  | FR1 | GAAAACTGCCGCACAACATTTAG |  | [5] |
|  |  |  |  |  |
| HRV | OL26 | CGGACACCCAAAGTAG |  | [6] |
|  | OL27 | CGGACACCCAAAGTAC |  | [6] |
|  |  |  |  |  |
| HCoV  (229E, OC43) | 229E-1 | GGTACTCCTAAGCCTTCTCG |  | [7] |
|  | 229E-2 | GACTATCAAACAGCATAGCAGC |  | [7] |
|  | OC43-1 | AGGAAGGTCTGCTCCTAATTC |  | [7] |
|  | OC43-2 | GCAAAGATGGGGAACTGTGG |  | [7] |
|  |  |  |  |  |
| HPIV  (1, 2, 3) | Para1 | CCTTAAATTCAGATATGT |  | [8] |
|  | Pr1 | GATAAATAATTATTGATACG |  | [8] |
|  | Para2 | AACAATCTGCTGCAGCAT |  | [8] |
|  | Pr2 | ATGTCAGACAATGGGCAAAT |  | [8] |
|  | Para3 | CTGTAAACTCAGACTTGG |  | [8] |
|  | Pr3 | TTTAAGCCCTTGTCAACAAC |  | [8] |
|  |  |  |  |  |
| FLU  (A, B) | AM149 | CTCATGGAATGGCTAAAGACA |  | [9] |
|  | AM501R | TGCTGGGAGTCAGCAATCTG |  | [9] |
|  | BM26 | TGTCGCTGTTTGGAGACACA |  | [9] |
|  | BM470R | TGTGATGCTTGTTTTTCGCA |  | [9] |
|  |  |  |  |  |
| HAdV | AV1 | GCCGAGAAGGGCGTGCGCAGGTA |  | [10] |
|  | AV2 | TACGCCAACTCCGCCCACGCGCT |  | [10] |
|  |  |  |  |  |
| HBoV^¶^ | NP1-F | GCACAGCCACGTGACGAA |  | [11] |
|  | NP1-P | Fam-TGAGCTCAGGGAATATGAAAGACAAGCATCG-Tamra |  | [11] |
|  | NP1-R | TGGACTCCCTTTTCTTTTGTAGGA |  | [11] |
|  |  |  |  |  |
| β-act^¶^ | β-act-F | CCCAGCCATGTACGTTGCTA |  | [12] |
|  | β-act-P | Fam-ACGCCTCTGGCCGTACCACTGG-Tamra |  | [12] |
|  | β-act-R | TCACCGGAGTCCATCACGAT |  | [12] |
|  |  |  |  |  |
| HBoV VP1 mRNA^¶^ | VP1-F | CTCACTTTTCAGACAAATATGTGGTTACT |  | * |
|  | VP1-P | Fam-TTCAGAATGGTCACCTCT-AMGBNFQ |  | * |
|  | VP1-R | TGTTGTTTCAATGGCCTCTGTT |  | * |

^¶^Real-time PCR; *Primers and probe designed for the present study.

1. Allander T, Tammi MT, Eriksson M, Bjerkner A, Tiveljung-Lindell A, et al. (2005) Cloning of a human parvovirus by molecular screening of respiratory tract samples. Proc Natl Acad Sci U S A 102: 12891-12896.

2. Zheng H, Peret TC, Randolph VB, Crowley JC, Anderson LJ (1996) Strain-specific reverse transcriptase PCR assay: means to distinguish candidate vaccine from wild-type strains of respiratory syncytial virus. J Clin Microbiol 34: 334-337.

3. Peret TC, Hall CB, Hammond GW, Piedra PA, Storch GA, et al. (2000) Circulation patterns of group A and B human respiratory syncytial virus genotypes in 5 communities in North America. J Infect Dis 181: 1891-1896.

4. Peret TC, Hall CB, Schnabel KC, Golub JA, Anderson LJ (1998) Circulation patterns of genetically distinct group A and B strains of human respiratory syncytial virus in a community. J Gen Virol 79 ( Pt 9): 2221-2229.

5. Falsey AR, Erdman D, Anderson LJ, Walsh EE (2003) Human metapneumovirus infections in young and elderly adults. J Infect Dis 187: 785-790.

6. Pitkaranta A, Arruda E, Malmberg H, Hayden FG (1997) Detection of rhinovirus in sinus brushings of patients with acute community-acquired sinusitis by reverse transcription-PCR. J Clin Microbiol 35: 1791-1793.

7. Kuypers J, Martin ET, Heugel J, Wright N, Morrow R, et al. (2007) Clinical disease in children associated with newly described coronavirus subtypes. Pediatrics 119: e70-76.

8. Echevarria JE, Erdman DD, Swierkosz EM, Holloway BP, Anderson LJ (1998) Simultaneous detection and identification of human parainfluenza viruses 1, 2, and 3 from clinical samples by multiplex PCR. J Clin Microbiol 36: 1388-1391.

9. Ruest A, Michaud S, Deslandes S, Frost EH (2003) Comparison of the Directigen flu A+B test, the QuickVue influenza test, and clinical case definition to viral culture and reverse transcription-PCR for rapid diagnosis of influenza virus infection. J Clin Microbiol 41: 3487-3493.

10. Hierholzer JC, Halonen PE, Dahlen PO, Bingham PG, McDonough MM (1993) Detection of adenovirus in clinical specimens by polymerase chain reaction and liquid-phase hybridization quantitated by time-resolved fluorometry. J Clin Microbiol 31: 1886-1891.

11. Neske F, Blessing K, Tollmann F, Schubert J, Rethwilm A, et al. (2007) Real-time PCR for diagnosis of human bocavirus infections and phylogenetic analysis. J Clin Microbiol 45: 2116-2122.

12. Nystrom K, Biller M, Grahn A, Lindh M, Larson G, et al. (2004) Real time PCR for monitoring regulation of host gene expression in herpes simplex virus type 1-infected human diploid cells. J Virol Methods 118: 83-94.
