# Supplementary material for: Detection of Human Bocavirus mRNA in Respiratory Secretions Correlates with High Viral Load and Concurrent Diarrhea
Source: PLoS One. 2011 Jun 20;6(6):e21083. doi: 10.1371/journal.pone.0021083 (PMC3118811; doi:10.1371/journal.pone.0021083)
Supplement: Table S2 — Clinical and demographic data of ARI patients (DOC) [file pone.0021083.s002.doc]

**Table S2**. Clinical and demographic data of ARI patients

| **Clinical Data** | **Patients** | | |
| --- | --- | --- | --- |
|  | **≤ 2 years old** | **> 2 years old** | **Total** |
| Number of patients | 745 (73.4%) | 270 (26.6%) | 1015 (100.0%) |
| Number of masculine gender | 410 (55.0%) | 125 (46.3%) | 535 (52.7%) |
| Age (median of months) | 5 | 63.5 | 8 |
| HBoV-positive | **40 (5.4%)** | **8 (2.9%)** | **48 (4.8%)** |
| HRSV-positive | 249 (33.5%) | 26 (9.6%) | 275 (27.1%) |
| Cough | 643 (86.3%) | 244 (90.4%) | 887 (87.4%) |
| Coryza | 361 (48.5%) | 172 (63.7%) | 533 (52.5%) |
| Sneezing | 134 (18.0%) | 93 (34.4%) | 227 (22.3%) |
| Fever | 455 (61.0%) | 208 (77.0%) | 663 (65.3%) |
| Wheezing | 340 (45.6%) | 80 (29.6%) | 420 (41.4%) |
| Dyspnea | 407 (54.6%) | 84 (31.1%) | 491 (48.4%) |
| Nasal obstruction | 259 (34.8%) | 116 (42.9%) | 375 (37.0%) |
| Diarrhea | 51 (6.8%) | 12 (4.4%) | 63 (6.3%) |
| Requirement for hospitalization* | 617 (82.9%) | 76 (28.1%) | 693 (68.3%) |
| Length of hospital stay (median of days) | 9 | 8 | 9 |
| Requirement for O_2_* | 386 (51.2%) | 40 (14.8%) | 426 (41.9%) |
| Requirement for PAP* | 66 (8.9%) | 6 (2.2%) | 72 (7.0%) |
| ICS = 0* | 84 (11.3%) | 142 (52.6%) | 226 (22.2%) |
| ICS = 1* | 91 (12.2%) | 59 (21.8%) | 150 (14.8%) |
| ICS = 2* | 160 (21.5%) | 28 (10.3%) | 188 (18.6%) |
| ICS = 3* | 123 (16.5%) | 15 (5.5%) | 138 (13.6%) |
| ICS = 4* | 130 (17.5%) | 16 (5.9%) | 146 (14.4%) |
| ICS = 5* | 104 (13.9%) | 5 (1.8%) | 109 (10.7%) |
| ICS = 6 | 32 (4.3%) | 1 (0.3%) | 33 (3.3%) |
| ICS = 7 | 21 (2.8%) | 4 (1.5%) | 25 (2.4%) |
| LRTI* | 622 (83.5%) | 99 (36.6%) | 721 (71.0%) |
| URTI* | 123 (16.5%) | 171 (63.3%) | 294 (28.9%) |
| AOM | 62 (8.3%) | 15 (5.5%) | 77 (7.6%) |
| GERD | 39 (5.2%) | 5 (1.8%) | 44 (4.4%) |

PAP=positive airway pressure; ICS= index of clinical severity; LRTI= Lower respiratory tract infection; URTI= Upper respiratory tract infection; AOM= Acute otitis media; GERD= Gastro-esophageal reflux disease. * p<0.05
