# Supplementary material for: Detection of Human Bocavirus mRNA in Respiratory Secretions Correlates with High Viral Load and Concurrent Diarrhea
Source: PLoS One. 2011 Jun 20;6(6):e21083. doi: 10.1371/journal.pone.0021083 (PMC3118811; doi:10.1371/journal.pone.0021083)
Supplement: Table S3 — Frequencies of detection of other respiratory viruses in HBoV positive and negative samples. (DOC) [file pone.0021083.s003.doc]

**Table S3**. Frequencies of detection of other respiratory viruses in HBoV positive and negative samples.

| Virus detection | Number (%) of samples | | |
| --- | --- | --- | --- |
|  | Patients with ARI  HBoV+ | Patients with ARI HBoV- | Patients without ARI |
| One single virus detected | 13 (27.1%) | 26 (54.1%) | 27 (54.0%) |
| >1 virus detected: | 35 (72.9%) | 13 (27.1%) | 0 (0.0%) |
| 2 viruses | 17 (35.4%) | 8 (16.6%) | 0 (0.0%) |
| 3 viruses | 13 (27.1%) | 5 (10.4%) | 0 (0.0%) |
| 4 Viruses | 2 (4.1%) | 0 (0.0%) | 0 (0.0%) |
| 5 viruses | 3 (6.3%) | 0 (0.0%) | 0 (0.0%) |
| Total | 48 (100.0%) | 48 (100.0%) | 50 (100.0%) |
