# Supplementary material for: Detection of Human Bocavirus mRNA in Respiratory Secretions Correlates with High Viral Load and Concurrent Diarrhea
Source: PLoS One. 2011 Jun 20;6(6):e21083. doi: 10.1371/journal.pone.0021083 (PMC3118811; doi:10.1371/journal.pone.0021083)
Supplement: Table S4 — Clinical and demographic data of patients in whom HBoV was detected alone or simultaneously with other respiratory viruses. (DOC) [file pone.0021083.s004.doc]

**Table S4**. Clinical and demographic data of patients in whom HBoV was detected alone or simultaneously with other respiratory viruses.

| **Clinical Data** | **Patients** | | |
| --- | --- | --- | --- |
|  | **HBoV alone** | **HBoV + other viruses** | **Total** |
| Patients | 13 (27.1%) | 35 (72.9%) | 48 (100.0%) |
| Masculine gender | 7 (53.8%) | 25 (71.4%) | 32 (66.6%) |
| Age (median of months) | 10 | 8 | 8.5 |
| Cough | 12 (92.3%) | 32 (91.4%) | 44 (91.6%) |
| Coryza | 9 (69.3%) | 17 (48.6%) | 26 (54.1%) |
| Sneezing | 2 (15.4%) | 6 (17.1%) | 8 (16.6%) |
| Fever | 8 (61.5%) | 19 (54.3%) | 27 (56.2%) |
| Wheezing | 5 (38.4%) | 15 (42.8%) | 20 (41.6%) |
| Dyspnea | 6 (46.1%) | 21 (60.0%) | 27 (56.2%) |
| Nasal obstruction | 5 (38.4%) | 10 (28.6%) | 15 (31.2%) |
| Diarrhea* | 7 (53.8%) | 3 (8.6%) | 10 (20.8%) |
| Requirement for hospitalization | 10 (76.9%) | 30 (85.7%) | 40 (83.3%) |
| Length of hospital stay (median of days) | 8.5 dias | 9 dias | 9 dias |
| Requirement for O_2_ | 6 (46.1%) | 17 (48.6%) | 23 (47.9%) |
| Requirement for PAP | 0 (0.0%) | 2 (5.7%) | 2 (4.0%) |
| ICS = 0 | 4 (30.8%) | 3 (8.6%) | 7 (14.6%) |
| ICS = 1 | 0 (0.0%) | 6 (17.1%) | 6 (12.5%) |
| ICS = 2 | 3 (23.1%) | 6 (17.1%) | 9 (18.8%) |
| ICS = 3 | 0 (0.0%) | 6 (17.1%) | 6 (12.5%) |
| ICS = 4 | 6 (46.1%) | 7 (20.0%) | 13 (27.0%) |
| ICS = 5 | 0 (0.0%) | 5 (14.3%) | 5 (10.4%) |
| ICS = 6 | 0 (0.0%) | 1 (2.9%) | 1 (2.1%) |
| ICS = 7 | 0 (0.0%) | 1 (2.9%) | 1 (2.1%) |
| LRTI | 9 (69.3%) | 32 (91.4%) | 41 (85.4%) |
| URTI | 4 (30.8%) | 3 (8.6%) | 7 (14.6%) |
| AOM | 1 (7.7%) | 3 (8.6%) | 4 (8.3%) |
| GERD* | 3 (23.1%) | 1 (2.9%) | 4 (8.3%) |

PAP=positive airway pressure; ICS= index of clinical severity; LRTI= Lower respiratory tract infection; URTI= Upper respiratory tract infection; AOM= Acute otitis media; GERD= Gastro-esophageal reflux disease.

* p<0.05
