# Supplementary material for: Detection of Human Bocavirus mRNA in Respiratory Secretions Correlates with High Viral Load and Concurrent Diarrhea
Source: PLoS One. 2011 Jun 20;6(6):e21083. doi: 10.1371/journal.pone.0021083 (PMC3118811; doi:10.1371/journal.pone.0021083)
Supplement: Table S5 — HBoV loads determined by real time PCR in nasopharyngeal aspirates. (DOC) [file pone.0021083.s005.doc]

**Table S5.** HBoV loads determined by real time PCR in nasopharyngeal aspirates.

| Sample | CT^¶^ | Viral load (copies/mL) | mRNA of HBoV | Co-detection with other viruses |
| --- | --- | --- | --- | --- |
| RP015/05 | 5.255 | 7.99E+13 | NT^#^ | - |
| RP034/05 | 21.67333333 | 1.91E+09 | NT | - |
| RP040/05 | 31.74666667 | 2.64E+06 | - | + |
| RP041/05 | 32.20333333 | 2.10E+06 | - | + |
| RP049/05 | 35.79666667 | 2.06E+05 | NT | + |
| RP054/05 | 16.01 | 1.07E+11 | NT | + |
| RP059/05 | 36.27 | 1.54E+05 | - | + |
| RP064/05 | 31.70666667 | 2.94E+06 | - | + |
| RP068/05 | 34.73666667 | 4.41E+05 | - | + |
| RP069/05 | 39.62 | 1.77E+04 | - | + |
| RP071/05 | 37.33666667 | 7.66E+04 | + | + |
| RP077/05 | 39.88 | 9.16E+03 | - | + |
| RP082/05 | 34.63666667 | 4.43E+05 | - | + |
| RP092/05 | 37.19333333 | 8.42E+04 | - | + |
| RP102/05 | 37.52 | 6.89E+04 | - | + |
| RP105/05 | 35.1 | 3.22E+05 | - | + |
| RP110/05 | 38.46 | 3.59E+04 | - | + |
| RP191/05 | 21.96 | 1.60E+09 | + | - |
| RP196/05 | 19.29333333 | 9.09E+09 | + | + |
| RP233/05 | 18.79333333 | 1.37E+10 | + | - |
| RP249/05 | 34.08 | 6.24E+05 | - | + |
| RP254/05 | 18.01 | 2.05E+10 | NT | - |
| RP257/05 | 35.42 | 2.66E+05 | - | + |
| RP272/05 | 17.65333333 | 2.64E+10 | + | + |
| RP276/05 | 33.12333333 | 1.16E+06 | - | + |
| RP062/06 | 31.55666667 | 3.02E+06 | - | + |
| RP071/06 | 21.74 | 1.75E+09 | NT | + |
| RP089/06 | 27.52666667 | 4.30E+07 | NT | - |
| RP128/06 | 24.06 | 3.97E+08 | - | - |
| RP162/06 | 39.15 | 2.34E+04 | - | + |
| RP239/06 | 33.22 | 1.10E+06 | - | + |
| RP245/06 | 27.2925 | 5.01E+07 | - | + |
| RP259/06 | 26.035 | 1.17E+08 | - | - |
| RP271/06 | 38.5425 | 3.46E+04 | - | + |
| RP312/06 | 14.5425 | 2.30E+11 | NT | - |
| RP058/07 | 37.015 | 9.25E+04 | - | + |
| RP145/07 | 26.92 | 6.40E+07 | - | + |
| RP164/07 | 37.48 | 7.41E+04 | - | + |
| RP219/07 | 16.085 | 7.16E+10 | + | - |
| RP271/07 | 37.8125 | 7.21E+04 | - | + |
| RP334/07 | 32.695 | 1.53E+06 | - | + |
| RP344/07 | 36.5225 | 1.31E+05 | - | + |
| RP357/07 | 27.7875 | 3.82E+07 | - | + |
| RP377/07 | 25.3675 | 1.66E+08 | + | - |
| RP386/07 | 19.3725 | 8.62E+09 | + | + |
| RP398/07 | 36.7025 | 1.22E+05 | - | + |
| RP429/07 | 18.205 | 1.82E+10 | + | - |
| RP440/07 | 23.47 | 6.19E+08 | + | - |

^¶^CT= Cycle threshold- number of cycles required for the fluorescent signal to cross the

threshold (ie exceeds background level) , ^#^Not tested,
